# Surgery

**Published:** 1899-11

**Authors:** John Parmenter, Nelson W. Wilson

**Affiliations:** Buffalo, N. Y., Professor of anatomy and adjunct professor of clinical surgery, Buffalo University Medical College; Buffalo, N. Y.


### SURGERY.

CONDUCTED BY JOHN PARMENTER, M. D., Buffalo, N. Y.,
Professor of anatomy and adjunct professor of clinical surgery, Buffalo University Medical College.

AND

NELSON W. WILSON, M. D., Buffalo, N. Y.

### THE ESSENTIAL REQUIREMENTS OF A MODERN OPERATIVE PROCEDURE.

DR. L. S. MCMURTRY, of Louisville, Ky., delivered the address on surgery at the Chicago meeting of the Mississippi Valley Medical Association, October 5, 1899, selecting for his subject the above title. Among other things he said that during the past fifteen years the province of surgery and its scope have been greatly enlarged. Many diseases and injuries that were formerly treated on the expectant plan and by palliative measures, with little hope of permanent relief or cure, are now cured by judicious surgical intervention. Notwithstanding the increase in hospital facilities in cities and towns, many important surgical cases must receive immediate attention at the hands of the family physician from the very nature and urgency of the disease or accident. Such cases are classified as emergencies and they include many major as well as minor injuries. When a major or minor surgical operation is to be performed certain fundamental principles of asepsis should be observed, and yet it is comparatively exceptional in private homes for minor surgical procedures to be carried out without the presence of pus following the operation.

The speaker said that there are three important steps to be observed: The first is the administration of the anesthetic. This requires skill, which can only be acquired by practice; hence, medical students should receive practical instruction in the administration of anesthetics. The second element of a surgical procedure consists in the control of hemorrhage. In the early days of abdominal surgery it was common to disregard the question of hemorrhage. All those present would remember the days when the drainage tube was more frequently used than it is now, and that within forty-eight hours after an operation it was customary to pump out considerable quantities of blood as the result of oozing from small vessels that were ignored during the operation. Nowadays, when more attention is given to hemostasis, hemorrhage is lessened on account of attention being directed to the small vessels. A third indication, and one to which the speaker devoted the remainder of his remarks, was the important one of asepsis. He gave a detailed statement of the most modern application of asepsis in the operating room and in the clinic, which was conspicuous all the way through for its simplicity.

---

THE USE OF GLOVES IN SURGICAL OPERATIONS.

SCHLOFFER (*Archiv. f. klin. Chir.*, Bd. lix., Heft 1; *British Medical Journal*) advocates the use of gloves in the performance of operations which do not demand much manual dexterity or delicate manipulation, and holds that such accessories are likely to be especially beneficial in the practice of surgeons who are unable through constant experience in antiseptic or aseptic methods, or through the absence of skilled assistance, to ensure absolute safety from infection. By the use of suitable and thoroughly disinfected gloves the operator may, in the first place, protect his hands when working on infected structures, against virulent pyogenic agents, and, in the second place, may prevent the transmission to a clean wound of an infective organism that in spite of a vigorous cleansing still remains on his fingers. The author reports favorably of the use in Wolfler's clinic of thin leather gloves, carefully disinfected at first by hot water, and afterwards by prolonged immersion in a 5 per cent. solution of lysol. The wearing of gloves, however beneficial such practice may be found, should not lead to any carelessness in disinfecting the skin of the hands, as it may be necessary in the course of an operation to work with the bare hand. It is stated that the use of operating gloves in Wolfler's practice has been attended with much success, and by a complete absence of those occasional failures that are met with after operations performed with careful observance of ordinary antiseptic or aseptic details.

